# Association of potential morally injurious events, moral injury and somatic symptoms of health in UK military veterans: a cross-sectional study

**DOI:** 10.1186/s40359-025-03788-5

**Published:** 2025-12-10

**Authors:** Gavin M. Campbell, Natasha Biscoe, Amanda Bonson, Dominic Murphy

**Affiliations:** 1 Combat Stress Centre for Applied Military Health Research, Leatherhead, Surrey UK; 2 King’s Centre for Military Health Research, King’s College London, London, UK

**Keywords:** Moral injury, Somatisation, Veteran, Military, PMIE, Armed forces

## Abstract

**Background:**

Moral injury can follow exposure to three types of potentially morally injurious event (PMIE). Both PMIE exposure and moral injury are associated with poor health and functioning outcomes. Moral injury, somatic symptoms and other impaired mental health and functioning outcomes are frequently observed as co-occurring in treatment-seeking military veterans. This cross-sectional study aimed to examine the associations between moral injury, somatic symptoms and other frequent co-morbidities, and the association between the PMIE type experienced and somatic symptoms. Better understanding the relationships between these comorbidities can help inform assessment and clinical interventions in complex and comorbid populations.

**Methods:**

A total of 428 UK armed forces veterans seeking treatment for mental health difficulties from a treatment charity completed measures exploring PMIE type, moral injury, somatic symptoms, and frequently co-occurring outcomes including anger, depression and anxiety, Complex PTSD (CPTSD) and PTSD. Single and multiple linear regression models were used to analyse the relationship between co-morbid symptoms. Logistical regression models were used to explore the relationship between PMIE type and somatic symptoms.

**Results:**

Linear regressions observed significant relationships between symptoms of moral injury and somatic symptoms, anxiety and depression, anger difficulties and CPTSD. Only associations with anger difficulties and CPTSD remained significant after adjusting for other significant health outcomes. Betrayal-PMIEs were significantly associated with somatic symptoms. Co-occurring anger was significantly associated with this relationship.

**Conclusions:**

These results indicate that moral injury symptoms appeared most strongly associated with co-occurring anger and CPTSD. Different PMIEs may result in differing somatic symptoms, with the presence of anger potentially playing a moderating role. This has significance for guiding deeper understanding of patient presentations, as well as indicating possible focus for transdiagnostic therapeutic interventions. The underlying mechanisms of moral injury including as a co-morbidity and its overlap with an array of health outcomes, require further exploration.

## Background

Military veterans may be exposed to higher rates and frequency of traumatic experiences and have been identified as having a higher prevalence of mental health difficulties such as post-traumatic stress disorder (PTSD), anxiety, depression, and problematic alcohol consumption compared to the civilian population [1, 2]. Military service may also involve exposure to potentially morally injurious events (PMIEs); experiences which are considered ‘high stakes’ and which threaten an individual’s deeply-held moral beliefs and sense of trust [3]. PMIEs typically include acts for which the individual perceives they are responsible or which the individual witnessed, and can be further categorised into acts of commission (e.g. causing harm to others), omission (e.g. failing to prevent harm to others), or betrayal (e.g. in which a trusted authority has behaved transgressively) [4, 5]. Following PMIE exposure, moral injury may subsequently develop. Although not currently considered a mental health diagnosis in its own right, moral injury is conceptualised as the enduring distress and maladaptive response(s) which can include profound feelings of guilt, shame and anger [6] in response to the individual’s moral pain or anguish.

As the understanding and conceptualisation of moral injury continues to develop [3], there is a need to explore how moral injury clinically presents in PMIE-exposed populations to further guide the development of both effective diagnosis and effective treatments.

### Complexity and co-morbidity in veterans

Mental health difficulties in treatment-seeking veterans rarely occur in isolation; clinical complexity and comorbidity is common [7] with subsequent implications for intervention selection and effectiveness. For example, PTSD is observed to be highly co-occurring with problematic anger in veterans [8], yet high levels of anger appear to negatively impact the effectiveness of gold-standard PTSD treatments [9]. Murphy and colleagues [10] have suggested that Complex PTSD (CPTSD) – a separate disorder to PTSD characterised by the presence of PTSD symptoms plus additional disorders of self-organisation (DSO) symptom clusters – may be more prevalent than PTSD in military veterans as the result of prolonged and repeated exposure to interpersonal trauma. Compared with PTSD, CPTSD appears to be more resistant to gold-standard treatments and more highly co-morbid amongst veterans [11]. Co-morbidities with psychological disorders may also include physical health conditions. Somatic symptoms such as joint and chest pain, dizziness and headache, can frequently co-occur in mental health treatment-seeking veteran populations, with implications for quality of life and the effectiveness of psychological interventions which typically do not attend to somatic distress [12, 13].

### Moral injury, co-morbidity and somatic symptoms

This complexity of psychological presentation can also include moral injury, which can also often co-occur with PTSD [14] and may share common symptoms, in particular the presence of negative cognitions and emotions [15]. Despite this symptomatic overlap, research suggests that moral injury is conceptually distinct; not all those who display moral injury meet criteria for PTSD [6, 16, 17]. Significant overlap may also be evident between moral injury and CPTSD. Currier et al. [18] found that in a sample of treatment-seeking veterans, those with CPTSD reported more severe symptoms of moral injury when compared to those with PTSD. Symptoms of moral injury in military veteran populations have also been linked with increased risk of depression, anxiety disorders, and suicidal ideation [6, 19, 20]. However, there is currently no gold-standard treatment for moral injury mental health difficulties [21]. Furthermore, effective evidence-based treatment for other co-occurring mental health difficulties such as PTSD, may inadequately address [22] or potentially exacerbate symptoms of moral injury [23].

Associations between symptoms of moral injury and somatic symptoms – such as chronic pain [24] – in veterans have also been observed, with those who endorsed symptoms of moral injury found to be significantly more likely to meet the threshold for high somatic symptom severity [25]. Therefore, a better understanding of the nature of the complexity of veteran clinical presentations which includes moral injury and somatisation is needed to help inform the development of more effective, transdiagnostic therapeutic interventions.

### PMIE exposure and somatic symptoms

Somatic symptoms may not be contingent on reporting symptoms of moral injury. Biscoe and colleagues [25] found that veterans who had experienced a PMIE but did not endorse symptoms of moral injury, reported more severe somatic symptoms compared to veterans who had not experienced a PMIE. This lends credence to the disaggregation of PMIE exposure from symptoms of moral injury when considering the possible links to somatic health in veterans, and provides tentative evidence that PMIE exposure alone may be associated with enduring somatic distress. Furthermore, there is nascent but mixed evidence that somatic symptoms may also be impacted by the specific type of PMIE experienced. Raney and colleagues reported that betrayal PMIEs specifically, but not perpetration or witnessed PMIEs, were associated with greater reported overall pain intensity amongst a sample of U.S. veterans [26]. Meanwhile, Hinkel et al. found that in a sample of treatment-seeking veterans with symptoms of moral injury, PMIEs perpetrated by the veterans and not those perpetrated by others were associated with chronic pain [24].

Central to these differences may be the salience of the specific emotional response resulting from PMIE exposure type. Perpetration PMIEs may result primarily in heightened guilt and shame, whilst those who have experienced betrayal-based PMIEs may be more inclined to experience anger as the overriding emotional response [27]. Guilt, shame and anger all cause different physiological responses in the body [28, 29]. Shame has been shown to lead to increased risk of compromised immunological response [30], guilt can increase the risk of chronic disease such as arthritis and cancer [31], and anger is associated with an increased chance of poorer cardiovascular health [32]. These findings suggest that PMIE types may be experienced differently physiologically, which in turn may endure and impact reported somatic symptoms and longer-term physical health outcomes.

However, the majority of these studies are of U.S. veteran cohorts who have distinct demographic, service and post-service healthcare experiences compared to other veteran populations [19] which may impact the associations between PMIE type and somatic symptoms. As such, the association between type of PMIE exposure and somatic symptoms in a treatment-seeking UK veteran population has not yet been explored.

### The current study

In light of the evidence of veteran complexity and co-morbidity, the aim of this study was to further explore the co-occurrence of moral injury and somatic symptoms with reference to other frequently reported co-occurring mental health outcomes in a sample of treatment-seeking UK veterans.

Furthermore, given that PMIE exposure may in itself be deleterious, the study aimed to understand for the first time any associations between specific PMIE type and the presence of somatic symptoms in UK treatment-seeking veterans. Whilst differences in associations were expected, no specific associations are hypothesised in light of the current mixed evidence.

## Methods

### Participants and ethics

This study employed a cross-sectional design. A randomised sample comprising 20% of the veterans who had sought help from a UK-wide veteran mental health charity were selected to take part. Those eligible had provided a contact email address; had previously consented to contact about research studies; and had attended one or more appointments for treatment at the charity in the previous 12-months. Of the initial 1,147 veterans included, 158 were removed due to invalid contact details, resulting in 989 veterans who were contacted and invited to complete online self-report measures. In total, 428 (43.3%) veterans completed the measures. Responders were more likely to be older (M_age_ = 50.5 years) compared to non-responders (M_age_ = 44.3 years). There were no other significant differences in sociodemographic or military characteristics. The sample was predominantly male (97.4%) and white (88.6%), with a mean age of 50.5 years (*SD* = 10.9). The majority (94.1%) reported they had served in a combat or combat-support role and had at least one deployment during service (86.4%). A minority (4.4%) left military service before completing four years of continuous service (Table 1). Further population details are reported by Williamson and colleagues [33]. Ethical approval was granted by the Combat Stress Research Ethics Committee (ref: pn2020) and was conducted in line with relevant guidelines and regulations including the principles of the Declaration of Helsinki. Informed consent to participate in the study was obtained from all participants prior to data collection. Data collection took place online between August and October 2020.

**Table 1:** Sample demographic and military characteristics

	Full sample M (SD)
Age (years)	50.5 (10.9)
*Gender*	*n* (%)
Male	417 (97.4)
Female	11 (2.6)
*Ethnicity*	
White	379 (94.8)
Ethnic minority	21 (5.2)
*Relationship status*	
Single, divorced, separated, widowed	266 (66.0)
In a relationship	137 (34.0)
*Employment status*	
Working or retired	223 (56.3)
Not working	173 (43.7)
*Housing status*	
No fixed address	36 (8.9)
Fixed address	367 (91.1)
*Role during military service*	
Combat/Combat Support	364 (94.1)
Non-Combat	23 (5.9)
*Deployed during service*	
Yes	370 (86.4)
No	58 (13.6)
*Military service of less than 4 years*	
Yes	17 (4.4)
No	368 (95.6)

*N* = 428; Percentages given are for valid data only. Total values may not sum to 428 due to missing data

### Measures

#### MIOS

The Moral Injury Outcome Scale (MIOS) [34] was used to categorise the PMIE and measure the severity of subsequent moral injury outcomes. The measure is split into two sections. The first section – PMIE Type – asks respondents to endorse which type of experience that *“went against your moral code or values”* has troubled them in the past month: one in which they “*did something or failed to do something”* (‘Self-PMIE’); one in which they “*saw someone or people do something”* (‘Others-PMIE’); or one in which they were “*directly affected by someone doing something or failing to do something”* (‘Betrayal-PMIE’). Respondents can endorse more than one category of PMIE. The second section – MIOS Outcomes Score – has 14-items which measure symptom severity inherent in moral injury. Respondents are asked to rate their agreement with statements such as *“I blame myself”*,* “I lost trust in others”* and *“I feel like I don’t deserve a good life”.* Statements are scored on a five-point Likert scale ranging from *“Strongly disagree”* to *“Strongly Agree”*. Scores are summed to provide a total MIOS Outcomes Score out of 56. No threshold score for meeting case criteria is set, but a higher score indicates greater burden of symptoms. The MIOS demonstrated good internal consistency in this sample (α = 0.95).

#### PHQ-15

Somatic symptoms were measured using the 15-item Patient Health Questionnaire (PHQ-15) [35]. A score of 15 and above out of a possible total of 30 indicates high somatic symptom severity that is of clinical importance and thus meets ‘caseness’. The PHQ-15 demonstrated good internal consistency in this sample (α = 0.86).

#### ITQ

Probable PTSD and CPTSD were measured using the 18-item International Trauma Questionnaire (ITQ) [36]. The measure is split into two sections. The first section features six statements attending to the three symptom clusters of PTSD (hyperarousal, avoidance and re-experiencing) plus three statements attending to PTSD symptom-related functional impairment in the past month. The second section features six statements attending to the three additional symptom clusters of CPTSD collectively referred to as disturbances in self-organisation (DSO; negative self-concept, interpersonal difficulties and affect dysregulation) plus three statements attending to DSO symptom-related functional impairment in the past month. Respondents are asked to score their agreement with statements on a five-point Likert scale ranging from *“Not at all”* (score 0) to *“Extremely”* (score 4), with a score of 2 or higher representing endorsement of that statement. Probable PTSD is indicated by endorsing at least one statement for each PTSD symptom cluster and endorsing at least one item relating to PTSD symptom-related functional impairment. Probable CPTSD is indicated by meeting the PTSD criteria plus endorsing at least one statement for each DSO symptom cluster and endorsing at least one item of DSO symptom-functional impairment. Participants are categorised as not having PTSD, having PTSD or having CPTSD, thus results are categorical. If a participant meets the criteria for CPTSD, they do not also receive a PTSD diagnosis. The ITQ demonstrated good internal consistency for the total scale, PTSD items and DSO items in this sample (α = 0.93, 0.85, 0.83 respectively).

#### GHQ-12

The 12-item General Health Questionnaire (GHQ-12) [37] was used as a measure of common mental health difficulties (CMD) including symptoms of anxiety and depression. A score of four and above out of a possible total of 12 indicates meeting probable case criteria for CMD. The GHQ-12 demonstrated good internal consistency in this sample (α = 0.90).

#### DAR-5

The five-item Dimensions of Anger Reactions scale (DAR-5) [38] was used to measure probable difficulties with anger. Possible total scores range from 5 to 20, with a score of 12 and above indicative of problematic anger difficulties. The DAR-5 demonstrated good internal consistency in this sample (α = 0.92).

#### SCI

Symptoms of poor sleep quality were measured on the eight-item Sleep Condition Indicator (SCI) [39], on which scores below 16 indicate possible disordered sleep. The SCI demonstrated good internal consistency in this sample (α = 0.91).

#### AUDIT

Alcohol use was recorded using the 10-item Alcohol Use Identification Test (AUDIT) [40], with a score of eight and above out of a possible 40 points indicating probable hazardous alcohol use. The AUDIT demonstrated good internal consistency in this sample (α = 0.98).

### Data analysis

This study was a secondary analysis [33]. Data were prepared in STATA 13.0 and analysed in SPSS v.26. Descriptive statistics were calculated for moral injury and each health outcome. Prior published analysis showed there were no significant differences in MIOS Outcomes Scores depending on military or demographic characteristics [25]. With the exception of MIOS Outcomes Scores, all measures were dichotomised into ‘case’ or ‘no case’ with reference to the stated threshold scores on each measure. Meeting case threshold was taken as indicative of clinically-relevant morbidity. Linear regression models were used to analyse the possible associations between moral injury and meeting case thresholds for co-occurring somatic symptoms and the other measured outcomes (e.g. CPTSD). In each model, moral injury MIOS Outcomes Scores were used as the predictor variable. An adjusted model using multiple predictor linear regression then controlled for each co-occurring outcome found to be significant in the single regression models. The sample met assumptions for multiple linear regression: the data were normally distributed (W = 0.99, *p* = 0.31) with low multicollinearity and a linear relationship between the variables used in the regression models.

The association between each of the three categories of PMIE Type and meeting case score thresholds for somatic symptoms was explored using single logistic regression models. The dataset also met assumptions for multiple logistic regression: the dependent variable in each model was ordinal level data; observations were independent; and independent variables were linearly related to the log odds of the dependent variable. The predictor variable in each model was PMIE Type (yes/no to endorsing each type of PMIE), and the outcome variable in each model was somatic symptoms (case/no case). ‘No’ was the reference category for each variable in these models.

Finally, in light of the clinical complexity and co-morbidity of treatment-seeking veterans, we explored whether co-occurring health outcomes (e.g. CPTSD) influenced any associations between PMIE Type experienced and somatisation. Post-hoc moderation analysis was used, incorporating any health outcome previously found to be significantly associated with moral injury symptoms. The influence was computed via multiple logistic regression models, with each PMIE Type, significant health outcome(s) (e.g. CPTSD), and their interaction term included as predictor variables, and somatic symptoms as the outcome variable.

A power analysis was not required as the study is a secondary analysis, was exploratory in nature and data were collected through convenience sampling with a fixed sample size [41].

## Results

### Moral injury, somatic symptoms and other health outcomes

The mean MIOS Outcomes Score was 34.0 (*SD* = 10.2) out of a possible score of 56. In total, 32.0% of the sample met the case threshold score for high somatic symptoms. Table 2 shows the mean MIOS Outcomes Scores on moral injury symptoms for those who did and did not meet score case thresholds for each health outcome measure. Higher MIOS Outcomes Scores were significantly associated with clinically relevant, high somatic symptoms (*R*^2^ = 0.04, *F*(1, 245) = 10.03, *p* = 0.02). In addition, reaching ‘caseness’ for CMD, anger difficulties, and CPTSD were also all significantly associated with higher MIOS Outcomes Scores in single predictor linear regression models (Table 2). In an adjusted multiple regression model controlling for each significant health outcome in the single predictor linear models (adjusted *R*^2^ = 0.26, *F*(4, 233) = 21.23, *p* < 0.001), only reaching case score thresholds for anger difficulties and CPTSD remained significantly associated with higher moral injury symptoms.

**Table 2:** Associations between moral injury symptom severity and caseness on co-occurring health outcomes

	Mean MIOS Outcomes Score	Unadjusted Model		Adjusted Model	
		β value	95% CI	Adjusted β^1^	95% CI
Somatic symptoms (PHQ-15)					
* No case*	32.39				
* Case*	36.53	4.25*	1.72–6.79	1.61	−0.81–4.03
CPTSD (ITQ)					
* No case*	27.25				
* Case*	37.20	9.84*	7.43–12.25	7.68*	5.04–10.33
PTSD (ITQ)					
* No case*	27.25				
* Case*	28.00	9.36	4.87–9.65		
CMD (GHQ-12)					
* No case*	29.16				
* Case*	34.71	5.93*	2.44–9.42	0.94	−2.42–4.31
Anger difficulties (DAR-5)					
* No case*	30.71				
* Case*	38.23	7.23*	4.80–9.65	4.99*	2.58–7.40
Sleep problems (SCI)					
* No case*	32.26				
* Case*	34.72	2.67	-0.13–5.47		
Hazardous drinking (AUDIT)					
* No case*	33.48				
* Case*	34.92	1.94	-0.70–4.57		

PTSD case excludes those who meet criteria for CPTSD, *PHQ-15* (15-item Patient Health Questionnaire), *GHQ-12* (12-item General Health Questionnaire), *DAR-5* (5-item Dimension of Anger Reactions), *SCI* (Sleep Condition Indicator), *AUDIT* (Alcohol Use Disorders Identification Test), β values for health outcomes in linear regression models with MIOS score as the predictor

^1^Adjusted for all other significant variables in table. The reference category for each health variable was not meeting case criteria for the disorder

*p < 0.05

### PMIE type and association with somatic symptoms

Across the full sample, 57.7% (*n* = 247) of participants reported exposure to a PMIE of any type. The three categories of PMIE were reported by approximately equal proportions of the sample: 60.3% reported experiencing Self-PMIE; 57.5% reported Others-PMIE; 57.1% reported Betrayal-PMIE. Participants could select more than one category. Table 3 shows the associations between PMIE Type and reaching case score threshold for high somatic symptoms. Betrayal-PMIEs were found to be significantly associated with somatic symptoms (χ^2^(1) = 18.17, *p* < 0.001). This association was not found for Self-PMIEs and Other-PMIEs.

**Table 3:** Associations between somatic symptoms and PMIE type

	*n* (%)	β value	95% CI	
Self-PMIE	149 (60.3)	0.01		−0.12–0.13
Others-PMIE	142 (57.5)	0.12		0.00–0.24
Betrayal-PMIE	141 (57.1)	0.26*		0.14–0.38

Percentages given are for population sample who reported PMIE exposure, respondents may select one or more categories. β values for PHQ-15 category as outcomes with PMIE type as the predictor in single logistic regression models, with ‘no’ as the reference category for PMIE type in each model. * *p* < 0.05

### Moderation analysis

Moderation analysis found that the interaction term between moral injury score (MIOS Outcome Score) and anger was not significantly predictive of somatic symptoms. However, moderation analysis of individual PMIE types found that anger difficulties were significantly moderating the relationship between Betrayal-PMIE and somatic symptoms (χ^2^(3) = 26.69, *p* < 0.001; β = 0.25, *p* = 0.04), indicating that greater comorbid anger symptoms increase the association between betrayal PMIEs and somatic symptoms.

## Discussion

In order to further understand the complexity of symptomatic presentations in treatment-seeking UK veterans, this study explored the co-occurrence of moral injury and somatic symptoms whilst considering other common co-morbidities. It demonstrated that somatic symptoms were significantly associated with more severe moral injury symptoms. This relationship was non-significant when controlling for other significant health outcomes, such as CPTSD. However, anger difficulties and CPTSD remained significantly associated with more severe moral injury symptoms.

The current study further explored the relationship between types of PMIE exposure and somatic symptoms. A difference in association was observed: the results suggest that only Betrayal-PMIEs were significantly associated with reporting somatic symptoms, irrespective of whether veterans reported symptoms of moral injury. Following the robust significant association found between both anger and CPTSD with moral injury symptoms, these co-occurrences were also explored with reference to type of PMIE exposure and somatic symptoms. Anger was found to play a significant role in the association between Betrayal-PMIE exposure and high somatic symptoms.

### The role of CPTSD

The enduring association of moral injury symptoms with CPTSD suggests that this co-occurrence may act as a confounder in the relationship with somatic symptoms and highlights the potential conceptual overlaps. Both moral injury and CPTSD share DSO symptoms as defining features [36] – namely emotional dysregulation, negative self-concept and interpersonal difficulties [42] – and moral injury symptoms have been found to have a large effect on the severity of reported DSO symptoms [43]. The present study tentatively further supports the finding of the importance of CPTSD symptoms in co-morbid and complex presentations of moral injury. Therefore, there is merit in further research exploring the nature of these associations [18] particularly with reference to the need for therapeutic interventions that are effective across multiple co-occurring disorders.

### PMIE type and somatic symptoms

Our finding that only Betrayal-PMIEs were associated with somatic symptoms is of note. A mechanistic explanation may be that veterans could potentially attribute their somatic symptoms to mistreatment and/or betrayal by others. Musculoskeletal disorders and injuries remain a leading cause of non-voluntary, medical discharge from the UK armed forces [44] and these may persist as somatic distress in veteran life. Therefore, it is plausible that for a significant number of veterans, medical discharges may be viewed as an institutional level Betrayal-PMIE.

However, it is also possible that acts of betrayal lead to inherently different outcomes in and of themselves. Significant associations between Betrayal-PMIEs, higher levels of depression, anxiety [45], probable PTSD or probable CPTSD [46] have been observed in healthcare workers. A more anthropological view suggests that somatic symptoms can be an idiomatic outward expression and communication of internal suffering [47]. As such, somatic symptoms may be a channel for outwardly communicating internal moral distress salient in Betrayal-PMIEs, in which personal trust has been violated [48]. This social distress signalling may act as a facilitator for promoting sympathetic or help-offering responses in others [49]. In contrast, it is possible that outward distress signalling for Self- and Other-PMIEs may be *less* desirable, in that it may invite undesirable scrutiny on the individual’s actions as part of the PMIE which are already a source of moral anguish.

### The role of anger after Betrayal-PMIEs

The association between severity of moral injury symptoms and anger, in addition to the potentially moderating role of anger in the relationship between experiencing a Betrayal-PMIE and somatic symptoms demonstrated in the current study, add to the developing evidence concerning potential differentiation of PMIE-type sequalae. For example, Bryan and colleagues [50] have suggested that Betrayal-PMIEs characterised by malign actions of another may be most readily suited to an predominantly externally-directed, dominant response such as anger. Evidence of this association is of clinical relevance. The presence of co-occurring anger or somatic symptoms may serve as a potential indicator of a veteran having potentially experienced a Betrayal-PMIE, thus informing clinician inquiry and appropriate treatment choices [21]. Anger in general has been characterised as the ‘forgotten emotion’ in military populations meriting specific therapeutic attention [51]. Therefore, considering and addressing the potential exposure to PMIEs – in particular those involving Betrayal – alongside presenting anger difficulties may be beneficial in creating effective, integrated interventions. Indeed, Morgan and Aldington [52] argue for an integrated approach to the understanding and treatment of co-occurring anger, somatic symptoms and possible moral injury. The associations between these outcomes found in the current study adds further credence to this approach.

### Strengths/limitations

A number of limitations must be acknowledged. Whilst demographically the sample is broadly representative of the UK veteran population in general [53], the findings may not extend to non-treatment seeking veterans or other populations such as civilians or different occupational groups. Furthermore, future attention is merited on gender and other minorities specifically in light of reported potential gender differences in symptomatic responses to traumatic exposure and health outcomes [54, 55]. The data was cross-sectional and therefore analysis of causal direction or changes in co-occurrence is not possible. It was not possible to state what treatments participants may or may not have completed before data collection.

The use of self-report measures in a convenience sample and inherent risk of bias and under-reporting in responses is acknowledged. Whilst the MIOS measure records the three types of PMIE, respondents can select any combination or number of PMIE type to describe their ‘most currently distressing’ experience [34]. A PMIE coming under two categories is ecologically valid - an individual could both witness (Other-PMIE) and also fail to intervene (Self-PMIE) in the same situation. In this respect, the MIOS does not permit the fine-grained discrimination of which category of PMIE may be the *most* salient to the individual’s distress, nor is it able to locate the PMIE to a specific time period such as a military service. Future studies should consider a further exploration of PMIE type, time and disaggregated outcomes.

## Conclusion

This study adds to and advances previously published results and explores the association between PMIE type, moral injury symptoms and somatic symptoms. An association was found between moral injury symptoms and somatic symptoms as an indicator of impaired physical health. However, this association was seen to be vulnerable when accounting for other significant co-morbidities. The persistent association of moral injury with CPTSD and anger shows that further exploration of these overlaps is required.

The novel finding that in UK treatment-seeking veterans only Betrayal-PMIEs were associated with somatic symptoms, demonstrates a difference in outcomes dependent on *how* an individual’s moral code has been challenged. The presence of anger acting as a potential moderator in this relationship, also provides tentative evidence of a possible clinical indicator for the potential presence of a relevant Betrayal-PMIE in a veteran’s life history which may require addressing. Further investigation of these co-occurrences in both veterans and other populations can provide the basis for advising diagnostic approaches, and developing effective, integrative interventions to help prevent and treat moral injury.

## Data Availability

The datasets used and/or analysed during the current study are available from the corresponding author on reasonable request.

## Abbreviations

PTSD: posttraumatic stress disorder
CPTSD: complex posttraumatic disorder
DSO: disturbances in self-organisation
PMIE: potentially morally injurious experience
CMD: common mental health difficulties
MIOS: Moral Injury Outcome Scale
PHQ-15: Patient Health Questionnaire
SCI: Sleep Condition Indicator
GHQ-12: General Health Questionnaire
AUDIT: Alcohol Use Identification Test
DAR-5: Dimensions of Anger Reactions scale
ITQ: International Trauma Questionnaire
